# Parental engagement in early intervention for infants with cerebral palsy—A realist synthesis

**DOI:** 10.1111/cch.12916

**Published:** 2021-11-01

**Authors:** Phillip Antony Harniess, Deanna Gibbs, Jeff Bezemer, Anna Purna Basu

**Affiliations:** ^1^ Physiotherapy Department Great Ormond Street Hospital London UK; ^2^ Institute of Education University College London London UK; ^3^ Children's Research Barts Health NHS Trust London UK; ^4^ Population Health Sciences Institute Newcastle University Newcastle upon Tyne UK; ^5^ Department of Paediatric Neurology Newcastle upon Tyne Hospitals NHS Foundation Trust Newcastle upon Tyne UK

**Keywords:** cerebral palsy, early intervention, infants, parental involvement, physical therapy

## Abstract

**Background:**

Emphasis on parental engagement strategies within occupational therapy and physiotherapy early intervention (EI) programmes for infants at high risk of cerebral palsy (CP) has increased. This reflects consensus that increasing parent participation enhances treatment efficacy, potentially improving infant and parent outcomes. However, evaluation of parental engagement in EI is complex. Despite the growing application of parental engagement strategies, aligned with family‐centred care practice, theoretical evaluation is currently lacking within the literature. This realist synthesis aimed to identify component theories underlying EI strategies to support parental engagement and to use empirical findings to evaluate how these work in practice.

**Methods:**

Realist synthesis: Databases Medline, Embase, Amed, CINAHL and PsychInfo were searched (from February 1985 ‐ February 2020); further articles were sourced from reference lists. A data extraction form was used, and a Critical Appraisal Skills Programme tool was used to assess study rigour.

**Results:**

Twenty‐six articles were included. Quality of relationships, parent education and intervention co‐design were the key themes related to parental engagement strategies. Findings indicate that constructive parent reasoning mechanisms of trust, belief, sense of control, perceived feasibility of home programme delivery and ultimately motivation are linked to the underlying intervention resources afforded by specific strategies (e.g., coaching pedagogy). These responses are precursors to engagement outcomes that include increased parental self‐efficacy and adherence. Importantly, parental self‐efficacy can initiate a process of change leading to improved parental confidence and anxiety.

**Conclusions:**

Sensitively designed programme strategies, centred on relational quality between parent, infant and therapist, are fundamental for effective parent connection, involvement and investment within EI for infants with CP.

**Key Messages**

- Collaborative early intervention parental involvement strategies require matching collaborative relational and communication styles for parents to rationalize trust.
- Co‐designed goals and home programmes (including educational materials) with realistic time commitments will help parents to believe in EI feasibility and benefits, creating increased adherence.
- Coaching and supporting parent‐infant sensitivity help parents to connect, participate and apply learning more effectively, whilst building outcomes of parental self‐efficacy
- Increased parenting efficacy may reinforce constructive parent reasoning responses (e.g., sense of control and motivation) to improve the underlying parental psychosocial context and sustain engagement long term.
- Stress may be an unavoidable parent response to involvement in early intervention (EI), particularly early on after neonatal trauma and adjusting to an uncertain diagnosis.

## 1 INTRODUCTION

Advances in developmental follow‐up assessments and clinical pathways has enabled earlier identification of infants at high risk of cerebral palsy (CP), in order to expedite referral to early intervention (EI) services (Novak et al., 2017). In this paper, EI describes targeted occupational therapy and physiotherapy (OPT) treatment for infants aged under 24 months, with understanding that intervention within this period could mitigate against the functional impact of a cerebral lesion at a time when neuroplasticity is heightened (Kolb et al., 2011; Morgan, Novak, et al., 2016). Such habilitation relies upon extensive repetition through trial‐and‐error opportunities (Hadders‐Algra, 2011). For example, learning to walk requires infants to take on average 2368 steps per hour with 17 falls per hour (Adolph et al., 2012). Constraint‐induced movement therapy (CIMT) also demonstrates the intensity that functional recovery requires (Gordon, 2011).

Focus on parental engagement within EI delivery has increased within a family centred care (FCC) framework, as opposed to therapist‐led child focused models of EI that have provided inconclusive outcomes in clinical research (Dirks & Hadders‐Algra, 2011; King & Chiarello, 2014; Morgan, Darrah, et al., 2016). Parental engagement shifts emphasis away from reliance on professionally dominated services, which are usually sporadic in nature, towards parents who have significantly more opportunities for interaction with their infants (Lord et al., 2018). Interventions with successful home translation by parents may promote greater therapy intensity, required for improved infant outcome (Mahoney & Perales, 2006). Outcomes of parental engagement in therapy delivery are not limited to infants, with potential benefits including parental learning and well‐being (Lord et al., 2018).

Conceptualizing parental engagement for evaluation is difficult. Adherence to intervention programmes is a relevant outcome but is only one component of engagement. Authors have described engagement as a co‐constructed process of ‘connection, involvement and investment’ (Mattingly & Fleming, 1994). Others have divided parental engagement into multiple components including affective (e.g., receptiveness), cognitive (e.g., willingness) and behavioural (e.g., self‐efficacy) involvement within OPT intervention which motivates work on tasks outside of sessions (King et al., 2019). Evaluation in this article will use this multiple component conceptualization and explore processes towards optimal states of parental engagement. Parental self‐efficacy (PSE), an example of one process, is defined as parents' belief in their ability to positively influence their child's development. However, parents of high‐risk infants are at risk of anxiety, stress and depression due to their infant's adverse neonatal experiences; this can disrupt the parent‐infant relationship (Aagaard & Hall, 2008; Benzies et al., 2013; Gibbs et al., 2015).

To date, systematic reviews in EI for infants with CP have mostly focused upon infant outcomes and are inconclusive regarding effectiveness (Hadders‐Algra et al., 2017; Morgan, Darrah, et al., 2016). These reviews select controlled trials; arguably, this design provides limited theoretical evaluation around causal processes in complex EI programmes to understand ‘what works, for whom and in what circumstances’ for optimal parental engagement (Autti‐Rämö, 2011; Pawson et al., 2005). This is likely the reason why, despite the growth of parental engagement description (with associated theory) in EI programmes, theory evaluation is lacking within the literature. Parent delivered intervention designs need well‐thought‐out development, clarity in implementation and sophisticated evaluation of inherent complexities, as they are embedded within open and changeable psychosocial and institutional contexts. A strong theoretical and empirical base is a prerequisite for exploring relationships between complex intervention components and outcomes (Kovach, 2009). Realist synthesis has the potential to inform EI programme development and is timely given the current gap in this field of literature.

### 1.1 Realist synthesis

This review followed realist synthesis methodology guidelines (Pawson et al., 2005; Wong et al., 2013). A realist synthesis seeks to develop explanatory theory by evaluating how programme mechanisms might work in given contexts to produce outcomes. A critical analysis of causal relationships is summarized within a context, mechanism, outcome configuration (CMOc) (Pawson et al., 2005). Context refers to elements outside the formal architecture of the intervention which influence mechanisms and outcomes. Mechanisms are considered as both the resources provided through an intervention and individuals' responses, positive or negative. Interventions rely on mechanisms becoming active to achieve their effects, via individuals' input. Outcomes, intended or unintended, are produced from how people respond to resources within their context (Pawson et al., 2005).

### 1.2 Review objectives

This review aimed to identify theories underlying EI strategies to support parental engagement with home delivered programmes; to synthesize empirical findings from the theories and to undertake a critical analysis of causal relationships summarized within a CMOc.

## 2 METHODS

### 2.1 Data sources

#### 2.1.1 Inclusion and exclusion criteria

A requirement for article inclusion was that OPT EI was provided after neonatal unit discharge (irrespective if also provided in hospital). Infants within studies were <24 months with CP diagnosis or prognostically ‘high‐risk’ of CP. Experimental studies were included where specific descriptions of parental engagement strategies were provided and where parent outcomes or experience were transparent for extraction, either quantitatively or qualitatively (e.g., process evaluation). Primary qualitative studies were included that explored parental experiences of OPT EI. Protocol papers were included with matched results papers (including feasibility studies).

#### 2.1.2 Resources searched

Five databases (Embase, Medline, Cinahl, AMED and PsychInfo) were searched using relevant keywords between 1985 and February 2020 (Table 1). This period provided a comprehensive range, encompassing historical changes in EI, rendering older literature irrelevant, in addition to advances in study quality. Terms ‘Involvement’ OR ‘Engagement’ OR ‘Participation’ were included in the original scoping searches but as these are not widely applied MeSH terms in this field, hits were restricted to below 50 across all databases. Therefore, to optimize coverage, these keywords were removed.

**TABLE 1:** Search strategy

Parent*.mp.
Caregiver*.mp.
Mother*.mp.
Father*.mp.
1 OR 2 OR 3 OR 4
Infant*.mp.
Baby.mp.
Newborn.mp.
Neonat*.mp.
Toddler.mp.
6 OR 7 OR 8 OR 9 OR 10
Physiotherapy.mp.
Physical therapy.mp.
Occupational therapy.mp.
Early intervention.mp.
12 OR 13 OR 14 OR 15
Cerebral palsy.mp.
5 AND 11 AND 16 AND 17
Limit 18 to (English language, human, peer review journal, infant/preschool age and year = ‘1985–2020’)

#### 2.1.3 Identifying primary sources

After the initial search, duplicates were removed, and then, titles and abstracts were screened using search criteria before full text review to produce the final list of included articles, for data extraction and synthesis. The inclusion/exclusion criteria were developed using a consensus team approach. Study selection was completed by one reviewer (PH) with an agreed process that any uncertainties could be referred back to the team for a final decision regarding inclusion. Ultimately, this was not required. Thematic development involved the team, whereby each author contributed to refine and check the validity of the results and the CMOc development.

#### 2.1.4 Data extraction and appraisal

Realist syntheses include studies with different designs including qualitative, quantitative and protocol papers; therefore, a bespoke data extraction matrix was created, which is summarized in Table 3. Methodological quality was evaluated using the Critical Appraisal Skills Programme tools: Rigour classification was assigned as poor, moderate or strong.

#### 2.1.5 Developing CMO configuration

For this review, ‘theory’ refers to proposed explanations for how given strategies were suggested to work in practice. Initial scoping for discernible programme theories, which focused upon parental engagement strategies, were extracted from articles; either from explicit descriptions (e.g., protocol) or, where undocumented, implicit theories were inferred from the intervention architecture. The initial iteration of proposed theories were placed into categories before the subsequent synthesis (Table 2). Using empirical studies, a further iterative process highlighted relevant themes around context, mechanisms and outcomes from parent experience or outcome data pertaining to affective, cognitive or behavioural domains of engagement in parent delivered programmes. These findings were evaluated against the categories of the initial proposed theories to build the synthesis, later conceptualized within the CMOc. The CMOc construction included several iterations from the identified themes, until a level of synthesis was reached that provided an explanatory interpretation of the combined primary data. This cyclical process was conducted by one reviewer (PH), with discussion and development of each iteration with the team until the final CMOc was agreed.

**TABLE 2:** Initial iteration of proposed theories

Theory one: Quality of relationships between parent, therapist and infant	Trusting and collaborative relationships between parents and OPTs are foundational for effective therapy co‐design and education (Gibbs et al., 2019). Supporting parent sensitivity with their infant's state regulation and behavioural cues, creates an enriched relational environment (attachment theory ‐ Bowlby), which supports infant stability whilst also facilitating keener observation of infants' for applied sensorimotor learning (Eliasson et al., 2016; Ohgi et al., 2004).
Theory two: Parent education	EI therapy education engages parents; delivered ideally within home, focus on parents learning to extend therapy provision through the family environment into daily routines (Basu, Pearse, Baggaley, Watson, & Rapley, 2017; Eliasson et al., 2016; Hielkema et al., 2010; Morgan et al., 2014; Palmer et al., 1990). Pedagogic strategy shifts to coaching; with aims to enhance families' coping strategies and autonomy development, in applying solution focused challenges to infant's development throughout family life (Eliasson et al., 2016; Hielkema et al., 2010). Programme curricula focus on applied neuromotor learning principles; inducing progressive self‐produced infant activity using ‘scaffolding’ theory (supported progressive learning), with appropriate toy choice and handling support that is reduced upon infant initiation (Eliasson et al., 2014, 2016; Morgan et al., 2014). Home programme (paper or video) provision supports parent learning (Basu et al., 2018; Dusing et al., 2018). Parent schedules (attentive to family constraints) or diaries foster focus and accountability (Campbell et al., 2012; Dusing et al., 2018; Hielkema et al., 2010; Morgan et al., 2014).
Theory three: Co‐designing intervention	Collaborative goal setting enables parents to prioritize meaningful goals for their family and guides treatment direction accordingly. Supporting parent participation, increases attention on therapy translation into daily routines (Eliasson et al., 2016; Hielkema et al., 2010; Morgan et al., 2014).

Mechanisms were differentiated into resources and reasoning, worked through the parent context (Dalkin et al., 2015). We propose resources are components afforded by an intervention which are introduced into a context to produce a desired response. Responses, often triggered on a continuum, are defined as parents' reasoning (i.e., beliefs and perceptions about resources) to initiate action.

## 3 RESULTS

### 3.1 Study design and methodological quality

**FIGURE 1:**
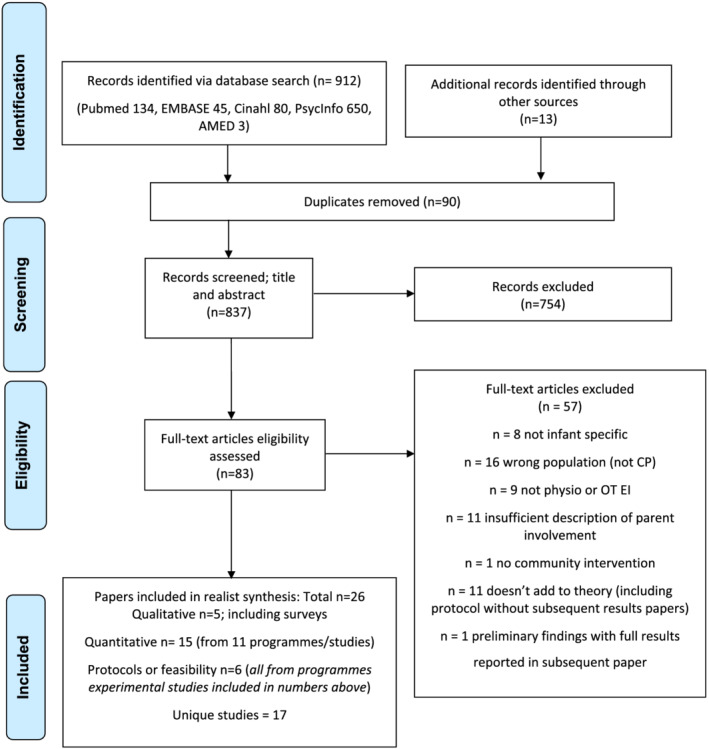
Literature search PRISMA flow diagram

Figure 1 summarizes the search process. Thirteen articles were identified through reference lists, with particular attention to recent systematic reviews (Hadders‐Algra et al., 2017; Morgan, Darrah, et al., 2016). Overall, six programmes were described in multiple papers (e.g., protocols, follow‐up results), comprising 15 of the 26 included articles. Table 3 summarizes the 26 included articles. A range of study designs were included; only three qualitative studies met the criteria. Many contemporary EI programmes provided transparency to facilitate realist inquiry, by including protocols and process evaluations.

**TABLE 3:** Summary of results

Programme (country), Authors, year Objectives	Study and programme description (population, setting and parent involvement intervention strategy)	Primary outcomes/findings	Design and methodological rigour	Key findings contributing to synthesis and theory refinement
**QUANTITATIVE STUDIES**				
Ballantyne et al., 2019 (CANADA) OBJECTIVE: TO DESCRIBE PARENTS' EXPERIENCES OF PRETERM INFANTS WITH CP AROUND TRANSITION TO COMMUNITY DEVELOPMENTAL REHABILITATION SERVICES FROM NEONATAL SERVICES	Canadian healthcare pathway for preterm infants with CP that move from neonatal services into a developmental rehabilitation service. (n = 18; 5 fathers)	Stress and anxiety experienced by parents is prolonged and is amplified when receiving diagnosis and transitioning between neonatal and rehabilitation. Fathers' experiences are subtly different to those of mothers, particularly around information seeking.	Parent interviews (62% telephone) – Thematic analysis Rigour: Strong. Triangulation for data validation and rigour. Limited reporting of reflexivity. Telephone interviews may have hindered contextual and nonverbal cues, probing and interpretation of responses.	Prolonged stress and anxiety from neonatal trauma shapes parental engagement with therapy healthcare services. Transitioning between health care providers is stressful and continuity is desirable for parents. Diagnostic experience stressful and creates grief. Therefore, parents seek information to make sense during uncertainty and engagement with HCPs initially focuses on this need.
EARLY THERAPY IN PERINATAL STROKE – ETIPS PROGRAMME (UK) Basu et al., 2017, 2018 TO CO‐DESIGN AND TEST FEASIBILITY AND ACCEPTABILITY OF ETIPS PROGRAMME	Feasibility study; parents of infants n = 27; 13 high risk of CP) Intervention co‐design (0–6 months age), acceptability and feasibility evaluation of programme that trains parents to deliver intervention targeting the affected side, with activities incorporated pervasively at home. Parents provided with materials including DVD and booklet. Guidance on infant self‐initiated activity on the affected side.	The intervention (including testing protocol) was acceptable to parents and practicable with daily routines. No adverse reactions were found to the treatment in the domains of parent well‐being and sense of competence.	Qualitative participatory co‐design focus groups, with one–one interviews and questionnaire. Rigour: Strong Achieved objectives of feasibility trial. High recruitment percentage (13/14), adequate sample with some attrition at 6 months (n = 2).	Co‐designed (parents and therapists) home programme information increases accessibility, supports realistic expectations with home routines and environment. This enhance acceptability and perceived feasibility of programme to parents. Higher quality of home education materials may elevate the importance of the information it contains for parents. Play oriented outcome measures support parent education around ‘scaffolding’ of activities. Play based therapy delivery supports positive parent‐infant interaction during treatment, encouraging deeper parental connection and involvement in therapy activities. Maintaining communication (e.g. email, text) with therapist between sessions reinforced involvement.
Broggi and Sabotelli, 2010 (USA) OBJECTIVE: TO INVESTIGATE (A) THE RELATIONSHIP THAT DEVELOPS BETWEEN PHYSICAL THERAPISTS AND PARENTS DURING EI AND (B) A POSSIBLE LINK BETWEEN THIS RELATIONSHIP AND EI OUTCOMES.	Parents n = 39 of children aged 9 months – 4 years, with motor delay (20% with CP). Used parenting stress index; measures of processes of care; percentage of goals achieved; family resources scale and satisfaction and control questionnaires. Analyses of association between relationship typologies and measures of parent stress, parenting competence and perception of service family‐ centredness.	Collaborative (FCC) relationships with therapists were perceived by parents to provide most satisfaction, control and reduced stress. Parents had high satisfaction with traditional (medical model ‐ didactic) relationships but a low sense of control. Distant relationships had the lowest level of satisfaction and control for parents.	Survey. Rigour: Poor. Small convenience sample and not representative; all mothers, no fathers and over 87% white background.	An intervention context of a collaborative parent‐therapist relationship offers parents an increased sense of control, which reduces their stress. Conversely parents may be satisfied with intervention contexts where more traditional medical, expert – Parent models are employed but their sense of control is not supported. This relationship may become discordant if challenged by parents. Parents may engage but stress will persist and engagement may wain. A low sense of parent efficacy and ceding to proxy expert control may foster dependence and lessen belief that they can build skills for independent carry over therapy actions at home.
Byrne et al., 2019 (USA) OBJECTIVE: TO EXPLORE PARENT AND HEALTHCARE PROVIDER PERSPECTIVES AND PRIORITIES UPON RECENT DIAGNOSTIC AND EI GUIDELINES (AS SET OUT IN Novak et al., 2017)	Parents (n = 17) and experts (n = 30) Exploration of Novak et al., 2017 guidelines for early detection and diagnosis of high‐risk of CP.	100% of parents stated early diagnosis or high risk for CP classification was beneficial compared with only 50% of providers who often gave early CP diagnoses before 12 months. Top parent priorities were honesty and positively phrased messages around prognosis.	Mixed methods Qualitative: World Café focus group workshops – Framework analysis in ICF Quantitative: Survey Rigour: Moderate. Framework analysis upon relevant guidelines. Relationships of researchers to participants not discussed.	Parents want early transparent and sensitive diagnosis of CP with clear prognostic information, which may aid parent adjustment and readiness for access and participation with EI services.
Campbell et al., 2012 (USA) OBJECTIVE: TO COMPARE KICKING AND TREADMILL TRAINING HOME PROGRAMME INTERVENTION IN ADDITION TO PHYSICAL THERAPY WITH BASIC PHYSICAL THERAPY	Intervention delivered by parents at home, from 2 months to 12 months CA, with monthly therapy visits. Infants n = 16, 7 in intervention group. Parents directed in home intervention protocol with infant kicking and treadmill training.	No significant differences between groups for infant motor outcomes Very poor levels of parent compliance with intervention protocol	Controlled trial. Rigour: Poor. Small sample. No randomization, convenience sample.	Distributed intervention model: Where access and support from a therapist is low (1x month) over a long period (10 months), even if minimal daily intervention input is required (12mins, 5 days per week), parent adherence will be low. This indicates therapy home visits provide support, accountability and focus for parents (e.g. diary completions increased prior to visits). Parent adherence decreased over time; highlighting challenges with maintaining engagement longitudinally.
COPING AND CARING FOR INFANTS WITH SPECIAL NEEDS ‐ COPCA PROGRAMME (NETHERLANDS) Hielkema et al., 2010 (PROTOCOL); Blauw‐Hospers et al., 2011 Hielkema et al., 2011; Dirks et al., 2016; Hielkema et al., 2019 OBJECTIVE: TO COMPARE COACHING BASED EI PROGRAMME VERSUS TRADITIONAL NEURODEVELOPMENT TECHNIQUES PHYSICAL THERAPY	Intervention duration of 3 months from 4 months CA; infants n = 46, 21 in intervention group Parent coaching to empower parent and family to lead and share treatment development by supporting the parent's own decisions through daily parent‐infant activities in home environment. Aims to encourage infant self‐directed motor activity with variation, using scaffolding ‘just right’ challenge principles.	Infant motor outcomes did not differ between groups. Process evaluation: Parent coaching using scaffolding strategies were factors associated with better infant motor and cognitive outcomes Traditional therapy hands‐on approach was associated with worse infant motor outcomes, particularly for infants with CP at 18 months.	RCT (Blauw‐Hospers et al, 2011; Hielkema et al., 2011; Dirks et al., 2016). Rigour: Strong. Robust description of intervention including theoretical foundations. Process evaluation fidelity to treatment and defined differences between ingredients of intervention and control arms in preliminary studies. Hielkema et al., 2019 Follow‐up questionnaires and interviews with families of original RCT. Rigour: Poor. Small sample size. Limited validity of psychometric tests in Dutch language. Approximately 80% questionnaire completion; due to study burden.	Parent coaching using scaffolding strategies to challenge active trial and error opportunities and promoting variation in daily routines were associated with better infant motor and cognitive outcomes. Coaching pedagogies may enable more optimal longitudinal parent engagement than traditional didactic therapy strategies. Parents of lower educational status benefit from coaching approach Psychometric parent stress measures do not differ between coaching and traditional therapy groups Coaching associated with increased family empowerment (parent autonomy and efficacy) and quality of life over time. Parents perceive less emotional worry and time restriction in relation to their child's additional needs
SUPPORTING PLAY EXPLORATION AND EARLY DEVELOPMENTAL INTERVENTION ‐ SPEEDI PROGRAMME (USA) Dusing et al., 2015, 2018 OBJECTIVE: TO EVALUATE FEASIBILITY (2015) AND EFFECTIVENESS (2018) OF A TARGETED INTERVENTION FOR INFANTS AT HIGH‐RISK OF CP (BRAIN LESION OR EXTREME PRETERM) AROUND TRANSITION FROM HOSPITAL TO HOME	Infants n = 14, 7 intervention (RCT) Two phase intervention. Ten sessions provided; 5 before discharge from neonatal unit and 5 at home. Parent education to provide daily input (20 minutes 5 day/week), including: Coaching around infant behaviour to identify appropriate treatment time; encouraging infant to self‐directed active exploration; providing ‘just right’ challenge and supporting posture. Educational materials provided; manual with pictures and videos of parent‐infant interactions.	Infants in the intervention group showed significantly more improvement in their exploratory/problem solving motor skills. No differences in ‘reaching’. Parent adherence to programme 120% of prescribed parent led intervention at home.	RCT (2018). Rigour: Moderate. Randomization and blinding of assessors. Protocol, supplemental materials and trial process evaluation (fidelity) provided transparency. Small sample size with loss of 3 to follow up, leaving study underpowered. In the feasibility study interviews the treating therapists appeared to be the same individual performing parent interviews (taken from parent quotes). However, despite parents reporting early challenges they did not evaluate parent stress quantitatively or qualitatively in the main RCT.	Families transitioning from neonatal unit may experience initial stress of participation in EI programmes. High adherence levels can still be sustained, by; Adequate support of the therapist (weekly), Accessible educational resources (8th grade educational level, including videos and photos), Realistic daily routines (scheduled diary of 20 minutes/5 days week), Time to adjust to programme expectations Observing benefits such as building understanding of infant. Pre‐existing family contexts of lower socioeconomic and educational status did not mitigate parent adherence levels. Greater parent preparation for EI home delivery by therapists on the neonatal unit could support parent readiness. Building early parent‐infant communication and play principles into programme aids parent sensitivity and awareness to their infant's development.
BABY‐CIMT PROGRAMME (SWEDEN) Eliasson et al., 2014 (PROTOCOL) Eliasson et al., 2018 OBJECTIVE: TO EXPLORE THE EFFECTIVENESS OF BABY‐CIMT (CONSTRAINT‐INDUCED MOVEMENT THERAPY) VS BABY MASSAGE FOR IMPROVING MANUAL ABILITY OF INFANTS WITH UNILATERAL CEREBRAL PALSY (CP)	CIMT with weekly physical therapist visits (1 hr) for 12 weeks; infants (<12 months) n = 37, 19 intervention) Parent delivered CIMT and therapy activities in home environment for prescribed time each day following weekly support from therapist, described as coaching approach.	Significantly greater improvements to motor function in affected upper limb activity for infants receiving CIMT. Enhanced sense of competence of being a parent among fathers in the baby‐CIMT group compared to fathers in the baby‐massage	RCT. Rigour: Moderate. Randomization and blinded testing process. Small sample with uneven comparison groups with attrition (n = 6), due to diagnostic predictive inaccuracy (resolving neuro signs or bilateral CP).	Parents found short daily intervention activities for infants (30 minutes, 6 days/week) with weekly therapy support feasible where infant's attention is limited and family life busy. This enabled high adherence (97%) to parent‐led home treatment programme. Parents (33%) found programme content difficult but perceived feasibility, adherence and satisfaction were high. Parents guided to encourage infants to be more active (rather than applying passive hands‐on interventions) and improvements attributed to this intervention created high satisfaction and increased fathers' sense of parenting competence.
Gibbs et al., 2019 OBJECTIVE: TO EXPLORE PARENTS' PERCEPTIONS OF EI THERAPY FOR INFANTS WITH EMERGING COMPLEX NEURODEVELOPMENTAL DIFFICULTIES.	6 mothers of infants with complex neurodevelopmental diagnoses (4/6 with CP) who had been admitted to a neonatal unit and were consequently receiving community EI therapy services within the same overarching health service for their child.	Four themes of parent experiences, described evolving relationships with therapy providers in the neonatal unit and following discharge: (1) a vulnerable start—Adjusting to the unexpected; (2) becoming a mother—Becoming a family; (3) the therapy journey; and (4) a new reality.	Qualitative 1–1 interviews, thematic analysis. Rigour: Moderate. Independent researcher (n = 3) coding for triangulation. Small sample within one local healthcare context limited scope and depth. Descriptive nature of categorization limited analytical depth.	Parent's belief in benefits of therapy provision for their child closely integrated with the interpersonal strength of the collaborative relationship with therapist. Parents adjust over time to a new reality with their child's emerging disability and advocacy for their child develops. Mothers faced many challenges Balancing family and work life with therapy commitments. Parents anticipated uncertain future but involvement in therapy enabled adjustment.
Holmstrom et al., 2019; Eliasson et al., 2016 (PROTOCOL) SMALLSTEPS PROGRAMME OBJECTIVE: TO EVALUATE SMALLSTEPS PROGRAMME WITH STANDARD CARE	Thirty‐nine infants at high‐risk of CP recruited at 4–9 months of corrected age (CA); n = 19 to small step group and n = 20 to SC. Intervention lasted 35 weeks; targeting mobility, hand use, and communication.	No difference between groups for infant outcomes. But infants worst affected within the intervention group catch up by the end of treatment.	RCT. Rigour: Strong. Clear randomization and blinding. Insufficient intervention‐control group differentiation; control used parent‐led therapy with family‐centred framework, so differences between interventions limited. Intervention group received 12 hours more therapy session hours. No reported parent‐led intervention hours at home.	No drop‐outs from the program and parents rated their motivation to engage in the program as very high (8.3 on a 0–9 purpose‐designed scale). Suggestive that coaching, collaborative goal setting and multi‐domain developmental therapeutic approach is important within intervention for optimal parent engagement.
Mattern‐Baxter et al. 2013 (USA) OBJECTIVES: TO COMPARE INTENSIVE HOME TREADMILL TRAINING WITH STANDARD CARE.	Duration 12 weeks intensive treadmill training; infants n = 12, 6 intervention. Parents directed in home treadmill protocol	Intensive treadmill training improved infant motor outcomes significantly more than standard care.	Controlled trial. Rigour: Poor. Small convenience sample with further sample attrition (n = 3).	Parents high adherence within this structured therapist directed programme, related to: 1, parents concern over infants' missed observable motor milestone (walking). 2, intervention intensive over short period, with focus on specific skill acquisition. 3, infant gaining observable skills related to parents' efforts through intervention. 4, skill development reduces burden of care.
GOALS AND MOTOR ENRICHMENT – GAME PROGRAMME (AUSTRALIA) Morgan et al., 2014, 2015; Morgan, Novak, et al., 2016 OBJECTIVES: TO COMPARE GAME PROGRAMME INTERVENTION TO STANDARD CARE	Intervention offered via home visits at least fortnightly from 4 to 5 months to 12 months CA; infants n = 30, 15 intervention (4 drop‐outs). Focus of collaborative goals with parents based on principles of active motor learning and environmental enrichment. Coaching approach suggested, but therapist guides intervention including provision of home programme.	GAME group significant gains in infant development and goal set outcomes vs standard care. Despite intervention group with more severe CP risk factors at baseline, younger infant age and parents with significantly greater depression scores. No significant difference in parent's perception of infant improvement between groups. Higher parent satisfaction with GAME programme.	RCT. Rigour: Strong. ‘TIDieR’ checklist used for intervention description. Clear randomization and single blind measurement. Although 4 dropped out of GAME intervention group. Mean age of the GAME group was four weeks younger than SC.	Collaborative goals developed (respecting an equal parent partnership, which acknowledges parents unique expertise and knowledge of their infant and home family environment) and regular therapy support and accountability (1x therapy session fortnight) brings outcomes of high satisfaction and adherence from parents (on average parents spent 20 minutes more a day). Parents' involvement in therapy does not increase or reduce levels of parents' depression levels.
Ohgi et al., 2004 (JAPAN) OBJECTIVES: TO COMPARE EI PROGRAMME (FOR INFANTS AT HIGH RISK OF CP) DIRECTLY SUPPORTING MOTHER INFANT RELATIONAL IN ADDITION TO PHYSIOTHERAPY VERSUS STANDARD CARE	Infants high‐risk CP, n = 23 (12 intervention) Intervention used neonatal behavioural assessment to support development of mother infant dyad within physiotherapy treatment. NDT techniques used. Sessions in hospital from neonatal unit to 6 months corrected age, every week.	There was significant decrease in mother's state anxiety. And confidence in caregiving score increased significantly in the treatment group. Infant motor outcomes improved more in the treatment group and were close to significance in an under‐powered study.	RCT. Rigour: Moderate. Good randomization processes followed. Small sample. Attrition in follow up data (31%), which was not accounted for satisfactorily. Descriptions of intervention arms were limited.	Support for parent‐infant relationship can help parents to understand their infant's individual behavioural characteristics better, allowing therapy delivery that maintains positive infant regulation facilitating better quality infant activity. This milieu could reduce parent anxiety and increases confidence leading to a deeper engagement in therapy. Parents with a higher educational and socioeconomic status may be a conducive context that increases receptivity to strategies supporting parent‐infant sensitivity.
Palmer et al., 1990 (USA) OBJECTIVE: TO COMPARE 12 MONTHS OF PHYSICAL THERAPY INTERVENTION WITH 6 MONTHS OF PHYSICAL AND 6 MONTHS OF PARENT‐TAUGHT DEVELOPMENTAL STIMULATION ACTIVITIES (LEARNING GAMES)	Infants with diplegia, n = 48; age 12‐19mths. Intervention group provided with 6 months of physical therapy and 6 months of parent taught curricula for infant stimulation (learning games) versus standard care of 12 months of physical therapy, biweekly sessions for both.	Infants that received 6 months of physical therapy and 6 months of learning games had more significant improvement to motor outcomes. Parent related outcomes e.g. parent‐infant relationship and home environment were not significantly different between groups.	RCT. Rigour: Strong. Randomization and blind assessment. Minimal attrition at 12 month follow up (n = 1) and good attendance to sessions (>90%).	Parent education of general infant development and incorporation of developmentally appropriate play is important within sensorimotor intervention curricula to promote infant and parental engagement. Introducing coaching around parent‐infant responsivity within the second year of life (within EI programmes) may not have as significant an effect upon parent‐infant outcomes, as providing this earlier within an infant's life.
Scales et al., 2007 (USA) OBJECTIVES: TO COMPARE PARENTS' PERCEPTIONS THE BENEFITS OF DIRECT INTERVENTION BY A PHYSICAL THERAPIST BENEFICIAL AGAINST AN APPROACH FOR GREATER PARENT INSTRUCTION	Parents, n = 23; 22 mothers. Comparison of parent involvement approaches. Parents observed videos of alternative therapy approaches within 2 sessions (i) therapist led hands, (ii) increased parental involvement. Then completed survey of their preferences.	Parents rated the parent instruction approach as more beneficial, but more stressful than direct therapist delivered intervention.	Survey. Rigour: Poor. Small sample and limited representation of different parent backgrounds. Survey was purpose designed for the study, with limited piloting and no psychometric testing prior to study.	Parents believed that training parents is more beneficial than therapist leading hands‐on intervention. However, parent involvement may also create a greater perceived burden and stress for parents (especially if sibling present). Parents believed that parental instruction approach would help the family and child to reach goals faster and would require less frequent physical therapy visits to achieve goals than direct intervention approach. Parents' educational status did not influence this perception.
Ustad et al., 2009 (NORWAY) OBJECTIVES: TO EVALUATE THE EFFECTS OF BLOCKS OF DAILY PHYSIOTHERAPY FOR INFANTS WITH CP	Infants high‐risk CP, n = 5, aged between 5 and 9 months. Therapist‐led treatment described as a standard eclectic physiotherapy EI approach using traditional hands‐on facilitative techniques in daily functional routines. Expectation for parents to carryover.	Compliance high, as measured by parents' sessional attendance. Parents preferred the intensive treatment blocks but reported increased stress and family‐life interruption due to high intensity. No difference in outcomes between intensive therapy and standard care.	Single‐subject design (ABABA). Rigour: Poor. Small sample and lack of comparison implicit to study design. Intervention model not feasible or transferable within a publicly funded healthcare system.	Some parents may prefer high‐intensity therapy intervention as it gives them greater opportunity to learn complex handling skills and transfer these into daily activities. Parents perceive expert therapy led handling treatment is more effective than their own and the least stressful option for them and their infant. But, this may lead to a greater dependence on therapy undermining parent self‐efficacy.

Abbreviations: CP, cerebral palsy; CIMT, constraint‐induced movement therapy; EI, early intervention; NDT, neurodevelopmental treatment; RCT, randomized controlled trial.

### 3.2 Intervention theories

Parental engagement theory and application has evolved within EI. Each iteration of initial proposed programme theory is summarized in Table 2, as ‘quality of relationships’, ‘parent education’ and ‘co‐designing intervention’. Table S1 provides the link between the original studies and these initial proposed theories. In the following section, the identified themes developed from the empirical studies are synthesized (using relevant findings) under the initial proposed theory. Table S2 provides a transparent overview of the process of connection of thematic development to the context, mechanisms and outcomes framework, including the derivation from empirical studies (using examples of raw data). The context, mechanisms and outcomes are integrated within the discussion as the programme theory is refined before summarizing within the CMOc.

## 4 SYNTHESIS: THEORY REFINEMENT

### 4.1 Theory 1: Quality of relationships between parent, therapist and infant

Trusting and collaborative relationships between parents and OPTs are foundational for effective therapy co‐design and education (Ballantyne et al., 2019; Gibbs et al., 2019). Supporting parent sensitivity with their infant's state regulation and behavioural cues creates an enriched relational environment, which supports infant stability whilst also facilitating keener observation of infants' for applied sensorimotor learning (Eliasson et al., 2016; Morgan et al., 2014; Ohgi et al., 2004).

### 4.2 Theory 1 synthesis

#### 4.2.1 Communication

Positive communication is fundamental for relational development. Parental willingness to respond to programme strategies is enabled or constrained by the therapeutic relational and communication approach (Gibbs et al., 2019; Holmstrom et al., 2019). Authentic verbal encouragement influences parenting confidence (Gibbs et al., 2019). Building collaborative therapeutic relationships generates parental reasoning of increased sense of control during difficult circumstances, reducing stress (Broggi & Sabatelli, 2010). Some parents may be satisfied with traditional directive relational styles (expert‐learner) but their unequal nature could restrict parent autonomy and subsequent PSE development (Hielkema et al., 2019). If parents challenge unequal dyads, discordant relationships could create disaffection and reduced receptiveness (Gibbs et al., 2019).

#### 4.2.2 Trust

Trust is a reasoning mechanism central to collaborative therapeutic relationships (Ballantyne et al., 2019). Trusting is a significant act for these parents who describe their circumstantial instability, therefore relational continuity is vital (Gibbs et al., 2019). Parental perceptions of therapists' authentic care for their infant and experience levels are important factors to therapists' trustworthiness (Ballantyne et al., 2019; Gibbs et al., 2019). Ultimately, trust underpins belief in the therapist, creating the milieu for mechanisms of parent belief in EI (Ballantyne et al., 2019; Gibbs et al., 2019).

#### 4.2.3 Greater parent‐infant connection

Greater parent receptiveness can be triggered through play and relational‐based therapy (Basu et al., 2017; Morgan, Novak, et al., 2016; Ohgi et al., 2004). If infants enjoy treatment participation through play, and activities are built on parental connection with their infant, then parents will invest more deeply in treatment (Basu et al., 2018).

Ohgi et al. (2004) specifically explored incorporating parent‐infant responsiveness training, meaning parents received support to observe and respond to their baby's behavioural communication cues during conventional physiotherapy. Infants receiving intervention demonstrated significantly less irritability, better state regulation and less stress; this connected to improvements in parent well‐being parameters such as reduced anxiety and increased confidence. In another study, parents felt that their observational skills were enhanced to see ‘tiny details’ of their infant's development (Dusing et al., 2015). When integrated into EI, together with appropriate play activities, parent sensitivity supported a greater two‐way enjoyment in the delivery, reinforcing parental connection (Holmstrom et al., 2019). However, introducing parent‐infant support later (18 months) is not as effective (Palmer et al., 1990). Therefore, attachment theory integrated early on (<6 months age) could support outcomes of increased parent sensitivity, responsiveness, PSE and decreased maternal stress and anxiety.

### 4.3 Theory 2: Parent education

EI therapy education is designed to engage parents in an active learning role. It is delivered ideally within home and focuses on parents learning to extend therapy provision through the family environment within daily routines (Basu et al., 2017; Eliasson et al., 2016; Hielkema et al., 2010; Morgan et al., 2014; Palmer et al., 1990). Pedagogic strategy now favours coaching, with aims to enhance families' autonomy development, in applying solution focused challenges to infant's development (Eliasson et al., 2016; Hielkema et al., 2010). Programme curricula focuses on neuromotor learning principles to induce progressive self‐produced infant activity using ‘scaffolding’ theory (supported progressive learning), with appropriate toy choice (Eliasson et al., 2014, 2016; Morgan et al., 2014). Home programme (paper or video) provision supports parent learning (Basu et al., 2018; Dusing et al., 2018). Parent schedules (attentive to family constraints) or diaries foster focus and accountability (Dusing et al., 2018; Morgan et al., 2014).

### 4.4 Theory 2 synthesis

#### 4.4.1 Readiness

Fundamental to effective education is parental readiness to engage. Yet the contextual backdrop means that circumstantial psychosocial challenges are foisted upon parents, including an emerging CP diagnosis for their baby following traumatic neonatal experiences and additional care burdens (Ballantyne et al., 2019; Basu et al., 2018). This can negatively impact parenting confidence, parent‐infant relationship development and engagement with therapy services by impinging on their affective, cognitive and behavioural involvement states (Gibbs et al., 2019). Adjustment processes to shock, grief and uncertainty are normal and are likely to continue unfolding beyond their child's infancy, with parents' initial engagement with therapists focusing on the need for information (Ballantyne et al., 2019). Parents overwhelmingly desire early, sensitive and transparent CP diagnosis disclosure, which is important for adjustment and grieving responses, although professionals are often reluctant to provide early diagnosis (Ballantyne et al., 2019; Byrne et al., 2019). Whilst processing the diagnosis, parental support networks (family, peer‐to‐peer and professionals) are critical for adjustment (Ballantyne et al., 2019; Gibbs et al., 2019). A research gap was identified around psychological therapies for parents at this point and specifically how this might influence engagement. Some argue that greater OPT support and preparation for families within the neonatal unit environment home transition could support readiness to engage in community EI (Basu et al., 2018; Dusing et al., 2015). Thereafter, parental engagement within EI itself can support adjustment (Ballantyne et al., 2019).

#### 4.4.2 Parent beliefs in educational resources

Parental beliefs around pedagogy are influential. Some parents perceive therapist‐led interventions as less stressful than parent participation in sessions, especially if siblings are present (Scales et al., 2007). Parent stress outcomes, as measured by psychometric assessments, do not differ between coaching and traditional therapy educational interventions (Hielkema et al., 2019). Nevertheless, parents believe their participation is more likely to make an intervention effective, whereas prolonged observer roles may create disengagement (Scales et al., 2007). Parents also believe that complex therapy handling techniques require substantial training (Ustad et al., 2009), which could undermine PSE development. However, coaching is associated with increased PSE over time (Hielkema et al., 2019).

Others have reported parental stress concerning engagement, particularly around transition to home from NICU and challenges in programme curricula (Basu et al., 2018; Eliasson et al., 2018), which may affect parents of lower socioeconomic status more (Dusing et al., 2015). Yet, within these programmes, parents still reported programme acceptability and feasibility, supported by high adherence and satisfaction. Co‐designed educational materials are vital to resonate with and motivate parents. Particular attention to language accessibility (pitched at 8th grade), use of photos and video, as well as the material quality (e.g., glossy manual), can increase parent receptiveness and learning (Basu et al., 2017, 2018; Dusing et al., 2018; Morgan, Novak, et al., 2016). In addition, activity focused play‐based outcome measures can positively support parent understanding, focus and connection, as planning ‘scaffolded’ activities becomes easier and fun (Basu et al., 2018).

#### 4.4.3 Adherence

Arguably, parent adherence signifies readiness and willingness, and is a useful (although limited) outcome measure of parental engagement. Adherence is higher when parents observe that infant milestones such as sitting or walking have not been met, presumably as greater awareness of the problem triggers motivation (Mattern‐Baxter et al., 2013).

Parent delivered home intervention may be perceived as a burden affecting adherence levels, with increased care demands of infants with CP particularly those more severely affected (Gibbs et al., 2019). Also, an infant's ability to engage within therapeutic activities is limited in early months (Dusing et al., 2015). Therefore, realistic expectations regarding parental time investment are necessary to make the programme workable. Some programmes have included daily time prescriptions for parent home activities, for example, 20 min 5 days per week (SPEEDI programme, [Dusing et al., 2018], 30 min 6 days per week Baby CIMT programme [Eliasson et al., 2018]) and 12 min 5 days per week (Campbell et al., 2012). Measuring against these prescribed times for parent led programmes, adherence (parent diaries) was above expected within the SPEEDI (120%) and Baby‐CIMT (97%), with parents reporting favourably upon their feasibility. Conversely, Campbell et al. (2012) found that despite lower expectations around daily parent input at home (12 min), adherence was poor with parents only able to complete 5 min, 2–3 times/week (average). The authors concluded the critical factor behind the low adherence was an inadequate number of supporting therapy sessions (once/month), in contrast to the SPEEDI and Baby‐CIMT programmes that provided weekly therapy sessions. This suggests that successful parent education that encourages adherence to parent delivered therapy relies on regular (i.e., weekly) face–face 45‐min sessions to support parental efficacy for adherence. Accessible communication with therapists is also important for families between sessions (Basu et al., 2018).

Long‐term adherence is another challenge. Campbell et al. (2012) found in their distributed intervention model that parent adherence regressed after 7 months of the 10‐month programme. Parental contexts including busy family life, multiple children and grief, may explain why long‐term home delivery is difficult to sustain in real life (Gibbs et al., 2019). Mass practice models (focused 3–4 month blocks) could allow parents to rationalize commitment, as discrete breaks allow normal family development particularly earlier in life (Dusing et al., 2015; Gibbs et al., 2019). Coaching, through enabling greater parent autonomy, is associated over time with reduced parental worry, reduced perceived time restriction and greater advocacy in relation to their child's additional needs (Hielkema et al., 2019). Therefore, coaching may support parental adjustment to diagnosis and create healthier parent perceptions of balancing time doing home therapy versus their own self‐care, facilitating parent well‐being and more sustainable long term engagement (Hielkema et al., 2019). Yet making causative assertions from such associations requires caution.

Self‐reported adherence data has clear limitations and does not appraise learning translation objectively. Only one study evaluated fidelity of parent delivery at home (Dirks et al., 2016). Within this coaching intervention parents translated learning to home, evidenced with video observations of providing progressive motor trunk activity during bathing, which was associated with improved motor outcomes.

### 4.5 Theory 3: Co‐designing intervention

Collaborative goal setting enables parents to prioritize meaningful goals and guides treatment direction accordingly. Supporting parental participation within the intervention design increases their attention on therapy translation into daily routines (Eliasson et al., 2016; Hielkema et al., 2011; Morgan et al., 2014).

### 4.6 Theory 3 synthesis

#### 4.6.1 Goals enhance parental engagement

Having goals and a clear plan during a time of uncertainty augments engagement, leading to greater adherence to parent delivery of home programmes compared to standard care for most parents (Morgan, Novak, et al., 2016). This is created by underlying cognitive mechanisms of belief in the intervention that are triggered by setting collaborative meaningful contextualized goals (Gibbs et al., 2019). If parental involvement is goal‐directed with time expectations, incorporating pervasive strategies and scheduling congruent to home routines, then the perceived burden will reduce, translating into greater belief in the workability of intervention thus furthering adherence (Basu et al., 2018; Dusing et al., 2018). Celebrating goal achievement can galvanize PSE and motivation, with retrospective reasoning as parents observe change following the intervention they provided and related reduced care burden (Basu et al., 2018; Eliasson et al., 2018; Gibbs et al., 2019; Mattern‐Baxter et al., 2013). Specifically, goal achievement builds fathers' PSE most effectively (Eliasson et al., 2018).

#### 4.6.2 Collaborative treatment planning

The GAME programme, versus standard care, demonstrated greater parental satisfaction and infant motor outcomes with focused collaborative goal and treatment planning, drawing the family home environmental context in to create an enriched motor programme (Morgan, Novak, et al., 2016). Collaborative treatment planning requires collaborative relationships, to increase motivation, enhance parental sense of control and reduce stress, whilst also creating a cognitive state of conviction that translation to home is possible (Broggi & Sabatelli, 2010; Holmstrom et al., 2019; King et al., 2019). Where parents' autonomous involvement is encouraged, it supports perceptions that their input is valued and validated by the therapist, therefore generating greater longitudinal engagement with programme resources (Hielkema et al., 2019; Morgan, Novak, et al., 2016).

## 5 CONTEXT–MECHANISM–OUTCOME CONFIGURATION

**FIGURE 2:**
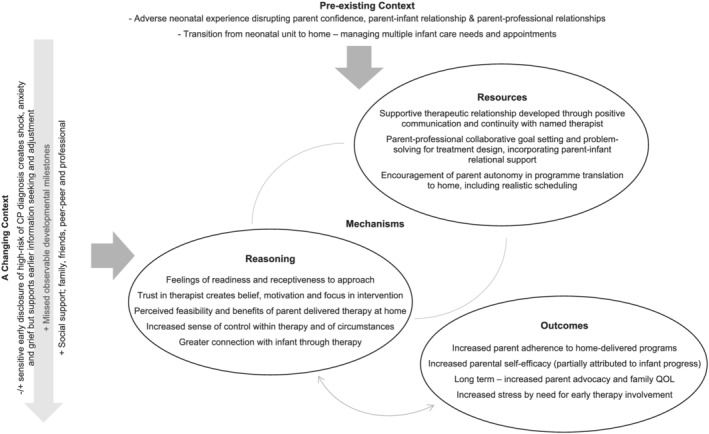
CMO configuration

The key findings of this synthesis are summarized as a CMOc (Figure 2) to portray the multifactorial contexts and mechanisms that have potential to influence parental engagement in OPT EI. Intervention strategies that induce positive parental mechanisms and create optimal outcomes of parental engagement are outlined in Table 4.

**TABLE 4:** Optimal intervention programme components

Optimal intervention programme components
Mass practice model: Entailing weekly therapy sessions 45–60 min in up to 12 week blocks
Relational continuity with named therapist
Educational coaching strategies within therapy sessions
Collaborative goal setting and contextualized home programme planning
Therapy is play‐based and provides parent‐infant relational support
Therapists are easily contactable between sessions

An important consideration is the interaction between mechanism resources and reasoning. Dalkin et al. (2015, p. 4) assert that resources and reasoning are ‘mutually constitutive of a mechanism’ but disaggregating them enables clearer differentiation between the mechanism and context, hence our use of their reworked CMO formula. This shows how resources afforded by the intervention, sensitive to the parental pre‐existing context, can work through these mitigating circumstances to create a positive response and produce desired outcomes. However, families are also living within a shifting context, which includes the adjustment of parents to an early CP diagnosis and increased awareness of the relevance of therapy due to associated missed motor milestones. This changing context can be highly influential, impacting particularly on parental reasoning within interventions, and therefore needs to be considered in relation to how the CMO interplay works. The outcome of stress (due to involvement expectations within intervention) during the first 6 months is unintended but unavoidable for some parents, due to multiple challenges at this time. Social support is an important in this parental context.

For clarity, we can apply an example of how one resource may initiate this CMOc loop. A coaching pedagogy supports parental autonomy in therapeutic activity translation and scheduling in the family home environment. Parents respond with reasoning, where they feel an increased sense of control and perceive feasibility in home delivery, subsequently generating outcomes including adherence and PSE.

Furthermore, evidence suggests that specified outcomes (e.g. PSE) could feed back to reinforce constructive parental reasoning (e.g. sense of control, trust and motivation). This may improve the parental psychosocial context, of low parenting confidence and anxiety related to post‐neonatal traumatic circumstances and adjustment to the CP diagnosis. Ultimately this could lead to more sustainable engagement, whilst positively influencing family QOL (Hielkema et al., 2019).

### 5.1 Parental socioeconomic and educational context

Many studies revealed parent demographic contexts where socioeconomic and educational status were below population averages (Dusing et al., 2018; Hielkema et al., 2011). Lower socioeconomic and educational status is often reported as a problematic context within early years' interventions (Cunha et al., 2018). Yet our synthesis indicates that it is not a deterministic factor around parental engagement but may elevate stress. Nevertheless, these referenced programmes have demonstrated that resources, sensitive to context (e.g. coaching and accessible educational materials), can induce parent responses of trust, belief, confidence and motivation leading to meaningful engagement outcomes, including adherence, even in the context of socioeconomic disadvantage.

## 6 DISCUSSION

This realist synthesis aimed to extract and evaluate theory underpinning parental engagement strategies within OPT EI literature. It found that resources such as supporting parental autonomy in home translation of therapy with a relational approach is afforded by specific strategies. Subsequent reasoning of increased trust, sense of control and motivation are critical intervening mechanisms that explain how multiple outcomes of engagement such as increased parental efficacy and adherence are created, regardless of parent background.

The literature indicates that coaching and parent‐infant support can create a deeper parent connection within EI whilst building PSE; however, by comparing to standardized care that have equally assimilated FCC principles, clear distinction is not always possible (Holmstrom et al., 2019). Improved coaching definitions in EI may help future differentiation (Akhbari Ziegler & Hadders‐Algra, 2020).

This synthesis builds an understanding of what parents believe is feasible in their involvement and time investment for home delivered programmes. Whether this ‘dosage’ provides sufficient intensity in infants for neuroplastic changes to ameliorate the functional impact of a cerebral lesion, is debatable. According to paediatric (ages 3–10) CIMT literature, a higher dosage (90 hours/15 days) versus lower dosage (60 hours/10 days) gives greater and longer lasting improvement (Gordon, 2011). Yet achieving these intensities with infants at high risk of CP is more challenging. Pervasive strategies may hold the key (Basu et al., 2018).

King and Chiarello (2014) argue that ‘family‐provider collaboration needs to be investigated at multiple points in the intervention process, because engagement and collaboration can change over the course of the intervention’ (p.1050). Our synthesis supports this assertion. Time is an important context where early home engagement can add stress, and long‐term adherence may also wane, particularly where support is insufficient. Sensitive balance is needed around parental adjustment to early CP diagnosis and readiness within EI. Therefore, the lack of research evaluating psychological support for parents during infancy is surprising (Irwin et al., 2019). Further research to understand the complexities surrounding parental states of readiness in EI (Hielkema et al., 2019) and embedding psychological support within programmes, could help understanding of how supporting parental adjustment might enhance affective components of engagement. In addition, research suggests that parent‐infant dyads are compromised in the first year, where there is a CP diagnosis (Festante et al., 2019). There is strong theoretical underpinning for parent‐infant support in contemporary programmes, with the logic that it mediates better outcomes in high‐risk infants and parents (Hutchon et al., 2019; van Wassenaer‐Leemhuis et al., 2016). Yet, more research is required to evaluate parental outcomes and causative links to motor developmental outcomes for infants with CP.

Our findings demonstrated that programme strategies were effective within contexts of lower socioeconomic and educational status but these families also reported early stress from their involvement. Other EI research for high‐risk infants shows infants of families with higher social risk may benefit most from intervention but at a greater cost to mental health (Spittle et al., 2018).

Education through collaborative communication with positive professional – parent partnerships is essential and parent programme adherence outcomes were promising in many contemporary programmes. Yet measuring parent adherence seems paradoxical to an equal and collaborative therapist‐parent relationship (Lawlor & Mattingly, 1998). Research is limited regarding what collaborative communication looks like in EI. One study observed parental engagement within EI analysing how parents reframe therapeutic education within their own embodied learning (Håkstad et al., 2018). Further research around parent and therapist engagement, collaborative communication and learning processes within EI could offer enlightenment for OPT education (Lawlor, 2012). Therapists would also benefit from understanding parents' individualized learning preferences to enhance resources afforded within intervention (Hurtubise & Carpenter, 2017). Ultimately, parents' motivation to learn through their active participation is interconnected with distinctive trusting therapeutic relationships, captured in related literature (Harrison et al., 2007; Hurtubise & Carpenter, 2017; Piggot et al., 2002).

Warnings exist within EI literature that good parent rapport and regular educational sessions may not always be sufficient for triggering parental adherence to home programmes, highlighting that teaching translation cannot be assumed (Badr et al., 2006). Yet fidelity evaluation of parent delivered home programmes presents from an acceptability, feasibility and ethical challenges. Parents may also feel guilt and stress if they perceive that they are unable to perform home programmes as effectively as ‘expert’ therapists (Dirks & Hadders‐Algra, 2011; Hinojosa & Andersen, 1991; Ross & Thomson, 1993).

### 6.1 Limitations

Only one reviewer performed the literature search, which may have implications for its rigour. However, the inclusion and exclusion criteria were agreed as a research team prior to the search, with extracted themes and CMOc development scrutinized as a team for validity and resonance.

Numerous studies were rejected for not providing parent data, despite their inclusion within other infant outcome focused systematic reviews (e.g., Hadders‐Algra et al., 2017). It was encouraging to observe diverse and representative samples, but we would advocate that research routinely captures parental outcomes, alongside infant.

The unique time under 24 months age within EI requires its own focus, hence papers for children between ages 2–4 years were excluded. However, a gap of parent perspectives of infants with CP aged <24 months exists, also highlighted by other reviewers (Kruijsen‐Terpstra et al., 2014), because previous practices provided CP diagnosis from 24 months. Nevertheless, our synthesis whilst applied in an EI context has transferability to other paediatric health/rehabilitation settings. For example, trust extension within parent‐therapist partnership is fundamental for all positive engagement outcomes within a family‐centred framework. Maintaining this equitable relationship requires open communication and frequent professional self‐reflection of their positionality (King & Chiarello, 2014; Lawlor & Mattingly, 1998). As infants become older the patterns of parental engagement become more established, emphasizing the importance of EI in creating foundational engagement expectations and outcomes that are sustainable for long‐term paediatric rehabilitation and parental well‐being.

## 7 CONCLUSION

This realist synthesis has teased apart critical mechanisms to explain how parental engagement can be optimized within OPT EI. It shows that parent reasoning responses of trust, belief, sense of control and ultimately motivation are linked to resources afforded by the programme, provided at a challenging time. Such parental reasoning is essential in creating engagement outcomes that include increased parental self‐efficacy and adherence. The findings provide valuable theoretical insights to further clinical practice and research in EI.

## FUNDING INFORMATION

This study is funded by the National Institute for Health Research, Integrated Clinical Academic Programme – Clinical Doctoral Research Fellowship, funders ref no. ICA‐CDRF‐2017‐03‐046 (Research Trainees Coordinating Centre). The views expressed are those of the authors and not necessarily those of the NHS, the NIHR or the Department of Health and Social Care.

## CONFLICT OF INTEREST

The authors declare that there is no conflict of interest.

## Supporting information


**Table S1.** Supplemental Table – Initial iteration of proposed theories audit trail

**Table S2.** Table: Context‐Mechanism‐Outcome Thematic Development from Empirical Studies

## Data Availability

Data sharing is not applicable to this article as no new data were created or analysed in this study.
