# Supplementary material for: Parental engagement in early intervention for infants with cerebral palsy—A realist synthesis
Source: Child Care Health Dev. 2021 Nov 1;48(3):359–77. doi: 10.1111/cch.12916 (PMC9298289; doi:10.1111/cch.12916)
Supplement: Supplementary file 1 — Table S1. Supplemental Table – Initial iteration of proposed theories audit trail [file CCH-48-359-s002.docx]

S1 Supplemental Table – Initial iteration of proposed theories audit trail

| **Key Programme Theories** | Theory description | Open codes | Examples of Raw Data |
| --- | --- | --- | --- |
| **Theory one: Quality of relationships between parent, therapist and infant** | - Trusting and collaborative relationships between parents and OPTs are foundational for effective therapy co-design and education (Ballantyne et al, 2018; Gibbs, Harniess, & Crossley, 2019). - Supporting parent sensitivity with their infant’s state regulation and behavioural cues, creates an enriched relational environment (attachment theory - Bowlby), which supports infant stability whilst also facilitating keener observation of infants’ for applied sensorimotor learning (Eliasson et al, 2016; Morgan et al, 2014; Ohgi et al, 2004). | Relational rapport building (therapist – parent, therapist – infant)  Parent-therapist collaboration  Parent – infant dyad support  Increased parent-infant sensitivity and observation in treatment | *“Developing rapport and forming a collaborative approach with the therapy team were important.”* (*p.263, Gibbs et al, 2019)*  *“Parents need help to modify their caregiving practices to enhance their infant’s development or to adapt to the infant’s abilities…The NBAS-based intervention was designed to facilitate the development of the infants by enhancing parenting skills and improving parental recognition of their infants’ abilities” (p.689 Ohgi et al, 2004)* |
| **Theory two: Parent education** | - EI therapy education engages parents; delivered ideally within home, focus on parents learning to extend therapy provision through the family environment into daily routines (Basu, et al, 2017; Eliasson et al., 2016; Hielkema et al., 2010; Morgan et al, 2014; Palmer et al., 1990). - Pedagogic strategy shifts to coaching; with aims to enhance families’ coping strategies and autonomy development, in applying solution focused challenges to infant’s development throughout family life (Hielkema et al., 2010; Eliasson, et al 2016).      - Programme curricula focus on applied neuromotor learning principles; inducing progressive self-produced infant activity using ‘scaffolding’ theory (supported progressive learning), with appropriate toy choice and handling support that is reduced upon infant initiation (Basu et al, 2017; Eliasson et al., 2016; Eliasson et al, 2014; Hielkema et al, 2010; Morgan et al., 2014). - Home programme (paper or video) provision supports parent learning (Basu et al., 2018; Dusing et al., 2018). - Parent schedules (attentive to family constraints) or diaries foster focus and accountability (Campbell et al., 2012; Dusing et al., 2018; Hielkema et al., 2010; Morgan et al., 2014). | Parent education for parent delivered intervention in home environment  Pedagogic scope: didactic - coaching  Contemporary programmes describe coaching  ‘Scaffolding’ sensorimotor learning with toys and handling  Learning materials provided  Scheduling home programme with parents  Keeping home programme diaries | *“Parents were trained in the daily home administration of the program, which was structured around checklists and specific behavioural objectives” (p.413 Palmer et al, 1990)*  *“…deliberate attention is paid to aspects of the home environment to enhance developmental outcomes.” (p.6 Morgan et al, 2014)*  *“Key words of [the] intervention are variation, exploration, trial and error, self-produced motor behaviour (no ‘hands on’), coaching (no training), family autonomy and family rituals” (p.3 Hielkema et al, 2010)*  *“…the training should be conducted at a level that is challenging, but not too demanding, i.e. the training should fall within the zone of proximal development, ZPD …The scaffolding and shared attentional focus provided by the ZPD aims to ensure that the child’s interest is maintained for as long as possible, which will promote the internalisation of independent developmental achievements.” (p.3 Eliasson et al, 2016)*  *“The materials comprised a pictorial manual tailored to the side of the stroke, a DVD with videos demonstrating the desired behaviours…” (p.3 Basu et al, 2018)*  *“Practice schedules are discussed and designed based on family time constraints.” (p.5 Morgan et al, 2014)* |
| **Theory three: Co-designing intervention** | - Collaborative goal setting enables parents to prioritise meaningful goals for their family and guides treatment direction accordingly. - Supporting parent participation, increases attention on therapy translation into daily routines (Blauw-Hospers et al, 2011; Eliasson et al., 2016; Hielkema et al., 2010; Morgan et al., 2014). | Collaborative goal setting  Treatment co-design with parents | *“In a goal-setting approach, the person’s own individual wishes are a strong ingredient, and it is suggested that the goals should be functional and meaningful for daily life” (p.3 Eliasson et al, 2016)*  *“On the basis of an ongoing, equal partnership in which the family defines the priorities for intervention…” (p.1327 Blauw-Hospers et al, 2011)*  *“…parents are encouraged to devise their own activities to enhance goal attainment” (p.6 Morgan et al, 2014)* |
