# Supplementary material for: Parental engagement in early intervention for infants with cerebral palsy—A realist synthesis
Source: Child Care Health Dev. 2021 Nov 1;48(3):359–77. doi: 10.1111/cch.12916 (PMC9298289; doi:10.1111/cch.12916)
Supplement: Supplementary file 2 — Table S2. Table: Context‐Mechanism‐Outcome Thematic Development from Empirical Studies [file CCH-48-359-s001.docx]

S2 Table: Context-Mechanism-Outcome Thematic Development from Empirical Studies

| **Context-Mechanism-Outcome** | Theme:   - Sub-Themes | Description | Examples of Raw Data | Study Source |
| --- | --- | --- | --- | --- |
| **Context** | Disruptive adverse neonatal experience:   - Home transition - Multiple care needs and appointments - Low parental confidence - Parent – infant connection - Adjustment - Parent – professional relationships - Infant attention /alert levels | Parents find transition from neonatal unit to home challenging. Neonatal trauma disrupts parental confidence, parent-infant interaction and parent-professional relationships. Higher demands of care activities (including more appointments) for parents creates stress and fatigue and interferes with EI treatment. This overall context may affect the readiness of parents to fully connect and invest in EI as required. | *“…parents described reliving their emotional trauma from the NICU”* (p.93 Ballantyne et al 2018)  *“Early family life was required to start in a very different setting than was anticipated, which could leave parents feeling separated from their babies and trying hard to find moments of connection. This period of adjustment continued after the baby came home.”* (p.262 Gibbs et al, 2019)  *“It’s not like you’re bringing a normal baby home, you’re bringing a sick baby home, or a baby with issues...that was a shock… I felt more like a nurse than a mum”* (Parent quote, p.263 Gibbs et al, 2019)  *““Sometimes multiple attempts were needed for the therapist to identify a time to provide the intervention when the infant was alert and could participate…some infants missed visits because of medical procedures such as surgery or eye examinations.”* (p.273 Dusing et al, 2015) | Ballantyne et al, 2018; Byrne et al, 2019; Basu et al, 2018;  Dusing et al, 2015; Gibbs et al, 2019 |
|  | Influence of early disclosure of high-risk CP diagnosis:   - Emotional response - Preferred earlier diagnosis with sensitive communication - Information seeking - Uncertainty - Adjustment - Awareness of missed milestones | Parents desire early diagnosis delivered in an honest and positive tone, which professionals may be reluctant to provide. Diagnosis creates emotional reaction of shock, grief and anxiety but also possibilities for information seeking and adjustment. Parents become more aware of their child’s delay when they miss observable motor milestones e.g. walking. | *“All parents described … how receiving their child’s diagnosis of CP was deeply emotional and overwhelming” (p.91, Ballantyne et al, 2018)*  Parent priorities include *“honest, realistic and hopeful”* early diagnostic disclosure of CP vs only 33% of professionals give high-risk of CP diagnosis (p.804, Byrne et al, 2019)  *“They [parents] want a discussion of CP specifically pertaining to their child”* (p.803, Byrne et al, 2019) | Ballantyne et al, 2018; Byrne et al, 2019; Mattern-Baxter et al, 2013 |
|  | Social support | Support networks including family, peer-peer and professionals enable parental adjustment. | *“Fathers uniquely described adopting the role of protector of their families. This role was characterized as gathering as much information as possible as a means of emotionally supporting”* (p.91 Ballantyne et al, 2018)  *“Mothers described their interactions with therapists as humanizing the transitions they were experiencing and saw them as an important means of support.” (*p.264 Gibbs et al, 2019)  *“Sometimes, parents experienced more comfort in seeking support from peers than from their family and friends”* (p.92 Ballantyne et al, 2018) | Ballantyne et al, 2018; Byrne et al, 2019; Gibbs et al, 2019; |
|  | Socioeconomic and educational status | A higher proportion of families with infants with CP have lower socioeconomic and educational status. | 50% of sample <$24,000 (poverty) \| 46% high school or less (Dusing et al, 2018)  72% maternal education low or middle (Hielkema et al, 2011)  67% of sample of higher social risk (Morgan et al, 2016) | Dusing et al, 2015; Dusing et al, 2018; Blauw-Hospers et al, 2011;  Hielkema et al, 2011; Hielkema et al, 2019;  Morgan et al, 2016 |
| **Mechanisms**  ***Resources*** | Parent Education:   - Coaching vs directive approaches - Sensitive progression for child - Quality educational materials | Educational approach considered as a resource to enable effective learning to enable effective parent-led home therapeutic activities in everyday life. Intervention studies use defined educational approaches with a strong emphasis towards coaching in recent studies.  ‘Scaffolding’ of infant self-produced movement individually applied within the home context. Home programmes are prominent and occasionally home schedules.  Educational pedagogies discussed with introduction of coaching strategies recently, with more or less theoretical support within given programmes. | *“It is known that parents can be effective treatment providers if they are properly trained and supervised”* (p.7 Eliasson et al, 2014)  *“Coaching of parents plays a prominent role in the Small Step Program. The aim is to provide a solution-focused approach that helps families to achieve goals that are unique and meaningful to them.”* (p.6 Eliasson et al 2016)  *“SPEEDI applies this theory by engaging parents in providing early experiences that are the “just right challenge” for the infant that day, matching the demand with the infant’s ability to support ongoing development”* (p.3 Dusing et al, 2018)  *“…parents … received an activity booklet … The booklet was written at an 8th grade educational level and had pictures demonstrating the activities”* (p.269 Dusing et al, 2015)  *“The materials comprised a pictorial manual tailored to the side of the stroke, a DVD and website with videos demonstrating the desired behaviours.”* (p.3 Basu et al, 2018) | Basu et al, 2017; Basu et al, 2018 Dusing et al, 2015; Dusing et al, 2018;  Eliasson et al, 2014; Eliasson et al, 2016; Hiekelma et al, 2010; Palmer et al, 1990 |
|  | Collaborative treatment planning:   - Collaborative goal setting - Collaborative problem solving - Encouragement of parent autonomy in home programme translation - Realistic scheduling | A collaborative approach between therapist and parent to develop goals and develop treatment plan for infant, fitting with family priorities and preferences while also respecting the parents’ expert knowledge of their child and home environment during implementation. | *“Parents were encouraged to provided activities daily, with a goal of at least 20 min per day of activities 5 days per week, provided by the parent… Parent was encouraged to develop a daily routine for encouraging developmental play*.” (p.3 Dusing et al, 2018)  *“As goals are attained the family and therapist work together to develop new goals … parent identified goal areas are targeted for practice during therapy sessions and built into a home programme”* (p.5 Morgan et al, 2014)  *“Parents are encouraged to use their knowledge of their child’s play preferences to elicit self-generated motor activity.”* (p.5 Morgan et al, 2014)  *“The program is also clear and specific within its different foci, with specific small achievable goals set in collaboration with parents, and strengthens the parental role by allowing parents to be in charge of the training situation.”* (p.14 Holmstrom et al, 2019) | Dusing et al, 2015; Dusing et al, 2018;  Eliasson et al, 2014; Eliasson et al, 2016; Hiekelma et al, 2010, Hiekelma et al, 2011; Holmstrom et al, 2019  Morgan et al, 2014 |
|  | Supportive therapeutic relationships between parent, therapist and infant:   - Positive communication - Continuity of relationship with therapist - Supporting transition home - Collaborative vs ‘traditional’ directive relational approach - Support for parent-infant relationship | The relational aspect between therapist, parent and child is a critical resource described in multiple studies, and is influential to all areas of intervention delivery such as treatment planning and educational approach. | *“One of the most significant elements to building a good relationship between parents and therapists was communication.”* (p.263 Gibbs et al, 2019)  *“The attitude of the therapist is crucial to training success, as the therapist must: a) support the parents’ sense of self-efficacy and confidence as treatment providers, so they can make the treatment situation enjoyable…”* (p.7 Eliasson et al, 2014)  *“… parents in this study described another distressing phenomenon, that of having to end a trusting relationships that was built over the first 2 years of their child’s life”* (p.95 Ballantyne et al, 2019)  *“(the physiotherapist) is one of the main constant people which I have, throughout the beginning to now. Nobody else has been so constant by our side.”* [parent quote] (p.263 Gibbs et al, 2019)  *“With preterm infants, there may be an advantage in starting parent training in eTIPS before the infant is discharged from hospital, to offset some of this burden.”* (p.11 Basu et al, 2018)  Relationship types; collaborative, traditional medical model, discordant or distant. *“…it is possible that increasing “either” satisfaction with the therapy/therapist “or” control over decision making may be sufficient to alter the outcomes experienced by families who express low satisfaction and low control.”* (Broggi et al, 2010)  *“Guided participation used in identifying cues to stop, alter, or delay interactions during caregiving, feeding, play activities”* (p.3 Dusing et al, 2018) | Ballantyne et al, 2019; Basu et al, 2018; Broggi et al, 2010; Dusing et al, 2015; Dusing et al, 2018; Eliasson et al, 2014; Gibbs et al, 2019; Ohgi et al, 2004; |
| ***Reasoning*** | Trust:   - Belief in therapist and therapy - Motivation and focus in intervention - Increased sense of control within therapy and of circumstances | Parents are able to reason that with involvement in the planning of home delivered intervention that they feel more in control. In a broader sense that they begin to feel more control over their difficult circumstances through their development by participating in the intervention. | “…*parents in the collaborative typology experienced less stress related to parenting and greater feelings of parental competence…*  *some families may prefer the relationship described by the traditional typology—as not all families wish to have significant control over decision making”* (p.243 Broggi et al, 2010)  *“…involvement with therapy services was a means by which they were gradually coming to terms with a re-visioned future of their child’s developmental progress.”* (p.265 Gibbs et al, 2019) | Broggi et al, 2010; Byrne et al, 2019; Gibbs et al, 2019; |
|  | Readiness | In response to the context of adverse neonatal experience and awareness of CP diagnosis parents will feel/ perceive their readiness to connect (emotionally and cognitively), involve themselves actively (behavioural) and invest in EI accordingly. Contextual factors outside of the immediate programme such as early disclosure of CP or support in transition home will enable or constrain parental feelings of readiness respectively. | *“At first it was hard until we got into a routine together, instead of on the nurses routine.”* [parent quote] (p.271 Dusing et al, 2015)  *“Based on their feedback we would see this as reflecting an increased need for support of these parents rather than a reason to exclude preterm infants from the intervention per se. With preterm infants, there may be an advantage in starting parent training in eTIPS before the infant is discharged from hospital, to offset some of this burden.”* (p.11 Basu et al, 2018) | Basu et al, 2018; Byrne et al, 2019; Dusing et al, 2015 |
|  | Parental beliefs in educational resources:   - Perception of approach - Connection/ receptiveness to training approach - Perceived feasibility of programme | Parents believe that their active involvement is beneficial to their learning, with support from the therapist. Intervention focused upon therapeutic handling skills is perceived to require more training. Coaching promotes more active and cognitive involvement of parents and connects to parental efficacy belief development.  The quality of educational content with attention to accessibility (including its co-design) is important to motivate and support parent learning. Parents perceive that the home delivered intervention is both achievable and effective, if it is delivered with sensitivity with realistic expectations according to their challenging circumstances. | *“… the majority of the participants preferred the therapist showing the parent how to assist the child during a play activity rather than the therapist doing “hands-on” intervention while the parent observed.”* (p.201 Scales et al, 2007)  *“[parents] reported that seeing a therapist frequently had given them the opportunity to really learn the handling skills. This had made the transfer into daily activities easier”* (p. 146 Ustad et al, 2009)  *“Maintaining this communication [email/text contact between sessions] also reinforced the importance…”* (p.8 Basu et al, 2018)  *“The parents… rated the program very high, i.e., above 8 on a 0–9 scale. This suggests that parents found that the different components… facilitated beneficial parental learning about the child’s overall development. The feasibility of Small Steps was also shown by the absence of drop-outs from the program and by parents rating their motivation to engage in the program as very high (8.3 on a 0–9 scale).”* (p.14 Holmstrom et al, 2019) | Basu et al, 2017; Basu et al, 2018; Dusing et al, 2015; Dusing et al, 2018; Hiekelma et al, 2019; Holmstrom et al, 2019; Morgan et al, 2016;  Scales et al, 2007; Ustad et al, 2009 |
|  | Greater parent-infant connection through intervention | Parents are able to develop a deeper connection with their child through the relational support of the intervention, use of play and a growing acceptance of their child’s disability through increased knowledge and understanding, which also creates increased confidence. | *“You realize certain things that you wouldn’t realize if you’re basically going on your own, but this (SPEEDI) will actually help you catch certain tiny details that you probably won’t see before.”* [Parent quote] (p.271 Dusing et al, 2015)  *“I never used to get smiles or anything like that. And I think all the eTIPS and stuff, and the playing, and stuff like that, and the different toys, I think that … I think, this has helped him interact with me*.” (p.8 Basu et al, 2018) | Dusing et al, 2015; Basu et al, 2018; Ohgi et al, 2004 |
| **Outcomes** | Increased parental adherence to home delivered programme.   - Performing home programme (self-report) - Fidelity to home programme | Parental adherence to home delivered programmes increases as a result of regular support and a positive response through reasoning such as belief, perceived feasibility and increased sense of control. | *“Parent documented a mean of 63.8 session (range 52–68) or 120% of the anticipated days of intervention.”* (p.7 Dusing et al, 2018)  *“Adherence to the study protocol was excellent … there were no drop outs … Baby-CIMT was conducted for a mean of 35 (SD 10) of the expected 36 h”* [97%] (Eliasson et al, 2016)  *“…immediately after the intervention, the infants of the COPCA group were significantly more often bathed in sitting position and less often in supine position than the infants of the TIP group (sitting: 77.7% vs 39.2%; median difference 32.0% (95% CI: 10.6–50.5)”* (p.152 Dirks et al, 2016)  *“Compliance with the protocol fell off even more in the latter part of the year… It is likely that … visits that only occurred monthly, was insufficient for maintaining adherence to the exercise program, especially for families with multiple life challenges.”* (p.23 Campbell et al, 2012)  *“The parents appreciated pauses in the intensive therapy…”* (p.146 Ustad et al, 2009) | Dirks et al, 2016; Dusing et al, 2018;  Eliasson et al, 2016; Mattern-Baxter et al, 2013; Ustad et al, 2009 |
|  | Increased parental self-efficacy (partially attributed to infant progress) | Parental self-efficacy (confidence, empowerment and self-belief) develops out of; attributing infant progress to the intervention they provide at home, a positive equitable relational and or coaching approach that promotes parental autonomy and mastery over circumstances and interventions where the parent-infant dyad is supported. | *“The PSCS revealed an enhanced sense of competence of being a parent among fathers in the baby-CIMT group compared to fathers in the baby-massage (p = 0.002).”* (p.191 Eliasson et al, 2018)  *“the total FES* [family empowerment score ~ self-efficacy]*, indicating that less time spent with the Neuro Developmental Treatment-approach and more time spent with the COPCA-approach was associated with better FES scores”* (p.4 Hiekelma et al, 2019)  Empowering caregivers aims to promote a sense of mastery over situations, which is positively related to psychological health of caregivers  *“The LCC score* [self-efficacy measure] *increased significantly in the EIP group. Mothers in the EIP group had a more positive perception of their parenting and childcare and judged their infants to be easier to care for.”* (p.693 Ohgi et al, 2004)  *“Mothers described overcoming challenges and becoming empowered in caring for their child.”* (p.265 Gibbs et al, 2019) | Eliasson et al, 2018;  Gibbs et al, 2019; Hielkema et al, 2019;  Ohgi et al, 2004; |
|  | Long term outcomes:   - Increased parent advocacy - Increased family quality of life - Reduced anxiety | Positive longitudinal outcomes may be associated with a coaching approach where there is an increase in family quality of life and reduced anxiety, as well as increased parental advocacy. | *“two other significant changes over time were present in the COPCA-group: the scores on “Impact emotional” and… “Impact time”…* *These increases imply that caregivers felt less emotionally worried and less restricted in time at the end of the intervention period than before the intervention had started.”* (p.6 Hiekelma et al, 2019)  *“in the COPCA-group caregivers’ quality of life increased significantly over time (T0: 8.1 (5.0–10.0); T3: 8.9 (6.8–10.0); p 0.004; HL 0.7 (95%CI 0.0–1.3))”* (p.6 Hiekelma et al, 2019)  *“The mothers’ “new reality” in our study indicated a place of acceptance as well as strengthened advocacy for their child in the face of fresh challenges.”* (p.266 Gibbs et al, 2019) | Gibbs et al, 2019; Hiekelma et al, 2019; |
|  | Increased parental stress for some parents through early involvement in intervention | An unintended outcome of parental involvement in some early intervention programmes may be increased stress. This may affect parents more during and immediately after the transition from the neonatal unit to home, and particularly parents who have a distant relationship with their therapist, which includes having less control over the treatment planning.  However, incorporating parent-infant support and collaborative relationships with therapist may mitigate for this stress in some parents. | *“A few mothers of preterm infants struggled with the overall burden of care for those infants due to other morbidity related to their prematurity”* (Basu et al, 2018)  *“…the type of [therapeutic] relationship experienced by families of children receiving EI is related to maternal stress, maternal competence, and maternal perceptions of family centeredness …”* (p.243 Broggi et al, 2010)  *“The STAI scores* [stress measure] *decreased significantly in the EIP group* [intervention using parent-infant dyadic support] *but did not change in the control group.”* (p.692 Ohgi et al, 2004) | Basu et al, 2018; Broggi et al, 2010;  Dusing et al, 2015; Ohgi et al, 2004 |
